# Self-assembly of photoresponsive azo-containing phospholipids with a polar group as the tail†

**DOI:** 10.1039/d0ra06803a

**Published:** 2020-09-04

**Authors:** Su Ma, Seiji Kurihara, Yasuhiro Tomimori, Sunnam Kim, Eunsang Kwon, Atsushi Muramatsu, Kiyoshi Kanie

**Affiliations:** Department of Applied Chemistry and Biochemistry, Graduate School of Science and Technology, Kumamoto University 2-39-1 Kurokami, Chuo-ku Kumamoto 860-8555 Japan kurihara@gpo.kumamoto-u.ac.jp; School of Chemistry, Biology and Material Engineering, Suzhou University of Science and Technology No. 99 Xuefu Road, Huqiu District Suzhou 215009 China; Graduate School of Science Research and Analytical Center for Giant Molecules, Tohoku University 6-3 Aramakiazaaoba, Aoba-ku Sendai 990-8578 Japan; Institute of Multidisciplinary Research for Advanced Materials, Tohoku University Katahira 2-1-1, Aoba-ku Sendai 980-8577 Japan kanie@tohoku.ac.jp

## Abstract

Vesicles or micelles prepared from amphiphiles with azobenzene (Az) moieties and long alkyl chains have attracted much attention in drug delivery systems. To induce release behavior from smart carriers *via trans*–*cis* photoisomerization of the Az groups, UV light exposure is typically used, but it can damage DNA and hardly penetrates cells. In this paper, Az-containing phospholipids without long alkyl tails were designed and synthesized; in these compounds, the end group of the Az moiety was substituted with a –NO_2_ and –OCH_3_ group (abbreviated N6 and M6, respectively). N6 self-assembled into H-aggregates with an interdigitated bilayered structure in water through the antiparallel orientation due to π–π interactions of the Az group, the attractive van der Waals forces, and the interactions and bending behavior of the phosphocholine groups. Vesicles showing visible light stimuli-responsive behavior were obtained by mixing N6 and M6, and the release of encapsulated calcein was triggered by visible light.

## Abstract 2

A mixture of a nitro- and methoxy-substituted azo-containing phospholipids without long alkyl tails formed vesicles showing visible light stimuli-responsive behavior. Release of encapsulated calcein from the vesicles was triggered by visible light.

## Graphical abstract

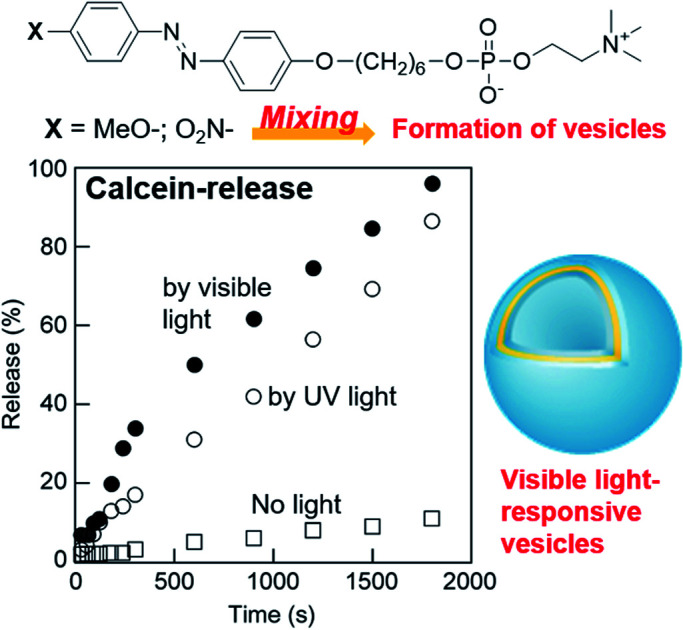

## 1. Introduction

Currently, in the field of drug delivery systems (DDSs), there is growing interest in developing smart carriers with various functionalities, such as pH-, electric-, magnetic-, temperature-, and light-sensitive carriers.^1–7^ Among them, light is regarded as the most desirable stimulus as it offers the unique properties of clean control, contactless perturbation and excitation tunability. Many reports are available on an azobenzene (Az), the conformation of which (linear *trans*- and bent *cis*-isomers) can be reversibly switched by exposure to specific wavelengths, triggering the release behaviour of light-sensitive carriers.^8–17^ Some such smart carriers with Az moieties have been developed into micelles or vesicles.^18–24^ Since micelles and vesicles are generally formed in water with amphiphilic compounds having both hydrophilic and hydrophobic moieties,^25–30^ much effort has been made to synthesize amphiphilic Az compounds to generate photosensitive micelles or vesicles.^31–33^ To the best of our knowledge, the reported amphiphilic Az compounds have generally one or two long alkyl chains because hydrophobic interactions among the long alkyl chains play an important role in forming self-assembling structures such as micelles and vesicles.^34,35,50,51^ In most DDS applications, photoisomerization of the Az moieties from the *trans*-form to *cis*-form upon UV light changes the hydrophilic-hydrophobic balance and molecular shape, which changes the morphology of the self-assembled structures, leading to the release of the loaded drugs. From the viewpoint of biological and medical applications, not only the precise molecular design of the stimuli-responsive self-assembling Az compounds but also the introduction of visible or near infrared light-responsivity to release the drug in the human body are required. For most Az compounds, *trans*–*cis* photoisomerization can only be induced by UV light irradiation; however, UV light is known to damage the human body *via* chemically altering DNA.^36,37^ Furthermore, UV light is strongly scattered, which makes it difficult for the light to penetrate into cells and tissues. Therefore, many efforts have been focused on Az compounds with photoresponsive groups sensitive to visible or near infrared light. In general, *trans*-Az compounds have two absorption bands, a strong π–π* transition in the UV region and a weak n–π* transition in the visible region. The π–π* transition of a push–pull type derivative of Az is in the visible region, enabling *trans*–*cis* photoisomerization upon visible light irradiation. However, the thermal reverse reaction of push–pull-type Az compounds from the *cis*-form to the *trans*-form is very fast.^38^ On the other hand, the introduction of electron-donating groups at the *o*- or *p*-position of Az compounds results in a redshift of the n–π* transition.^39–42^ The absorption coefficient of the n–π* transition is generally small, so intense light is required for *trans*–*cis* photoisomerization. To use Az compounds in photosensitive smart carriers, it is important to improve their sensitivity to visible- or near infrared light. In the case of the aforementioned micelles and/or vesicles composed of amphiphilic Az compounds, the features contributing to the self-assembly and the photoresponsive properties are distinct: the long alkyl chains and the Az moiety. The previously reported amphiphilic Az compounds that self-assemble to form micelle and/or vesicle structures generally have one or two long alkyl chains. The long alkyl chains play an important role in self-assembly through hydrophobic interactions. The Az moiety is responsible for the photoresponsiveness of the micelles and/or vesicles. Throughout the UV light-induced photoisomerization of Az, the strength of the hydrophobic interactions among the long alkyl chains that are responsible for the self-assembled structures remains almost constant. Therefore, the photoresponse in the morphology of the self-assembled structures is dominated by the relative relationship between the strength of the hydrophobic interactions among the long alkyl chains and the perturbation effect induced by the photoisomerization of the Az groups. Undoubtedly, a photoinduced morphological change in smart micelles or vesicles can be observed if the driving force for the self-assembly is diminished by photoisomerization.

Recently, Lee *et al.* reported that Az groups form π–π stacking interactions when phenyl derivatives with tris-Az groups are dissolved in THF–water mixtures, and this driving force led to self-assembling behaviour in which the molecules stacked into vesicles.^43^ The morphology of the vesicles was considerably destroyed upon irradiation, which was a result of the photoisomerization of the Az moieties and the total disruption of the interactions among the molecules. According to that study, the π–π stacking of Az groups is a desirable strategy for preparing photosensitive carriers for DDS. The morphology of the Az aggregates could be effectively controlled because the π–π stacking is closely related to the planarity of the Az groups, which can be easily adjusted through photoisomerization. Notably, the molecular orientation in the π–π stacking can be controlled by dipole–dipole interactions, as reported by Tang *et al.*^44^

**Scheme 1:**
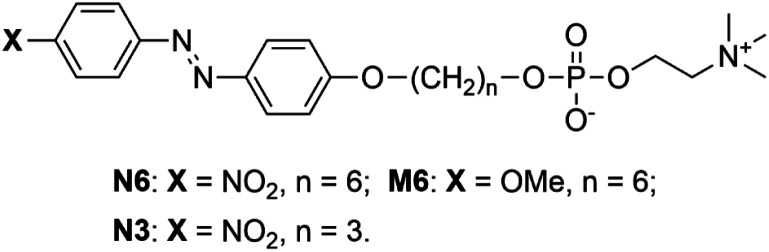
Phospholipids having an Az group; N6, M6, and N3.

In this study, we designed and synthesized Az-containing amphiphilic phospholipids without long hydrophobic alkyl tails. Instead, a nitro or a methoxy group was present at the end of the Az group. The phospholipids with nitro and methoxy groups with different alkyloxy chain lengths are abbreviated as N6, M6, and N3 (Scheme 1). The phospholipids were synthesized in a manner similar to that reported earlier.^45^ The chemical structures and purities of phospholipids N6, M6, and N3 were determined by ^1^H NMR measurements and elemental analysis. Az phospholipid N6 formed an H-aggregate with a bilayer structure at high concentrations in water. X-ray single crystal analysis of N3 revealed that the N6 molecules were ordered in an antiparallel orientation due to π–π interactions and their large dipole moments. Furthermore, the formation of self-assembled vesicles in water was observed upon mixing N6, which has a large dipole, and M6 which has no dipole. To investigate the photosensitivity of the vesicles that were spontaneously formed through the self-assembly of N6 and M6, encapsulated calcein was used as a probe.^46^ The calcein-encapsulating vesicles exhibited photoresponsive behaviour. In this study, we will discuss the self-assembly of Az phospholipids without a long hydrophobic alkyl chain at the end of the molecule and the photoresponsive behaviour of the self-assembled structures in water with respect to their morphological changes. The result was very impressive in the field of smart DDSs in response to light triggers.

## 2. Experimental

### 2.1 Materials

All reagents and solvents were of the highest commercial quality (Sigma-Aldrich Chemicals, FUJIFILM Wako Pure Chemical Co., Kanto Chemical Co., Inc., or Tokyo Chemical Industry Co., Ltd.), and they were used as received. Water was doubly distilled and deionized prior to use. All of the organic reactions were carried out under an argon atmosphere in dry solvent. Glass vessels for the organic reactions were well dried by heating under reduced pressure. Progress of the reactions was monitored by thin-layer chromatography using 0.25 mm E. Merck silica gel plates (Silica Gel F254), and the plates were visualized with UV light and/or dipping the plates in ethanolic sodium phosphomolybdate followed by heating. Purification was carried out with a Yamazen Fast Flow Liquid Chromatography system using high-flash columns (SiO_2_ followed by ODS). Phospholipids having an Az moiety (N6, M6, and N3, shown in Scheme 1) were synthesized in a manner similar to that reported earlier.^45^ The chemical structures and purities of phospholipids N6, M6, and N3 were determined by ^1^H NMR measurements and elemental analysis (see, ESI†).

### 2.2 Self-assembly behaviour of N6/M6 mixtures in water

To completely dissolve N6 and M6 in water, aqueous suspensions containing N6 or M6 at various concentrations were heated to 90 °C. Then, different volumes of the resulting hot solutions were mixed to adjust the molar ratios of N6/M6 to values from 0/100 to 100/0. After cooling to room temperature, the N6/M6 mixtures in water were used in the following experiments. The self-assembly behaviours of amphiphilic phospholipids with an Az unit in water were investigated by measuring the surface tension and the absorption spectra. The surface tension and absorption spectra were measured with a SITA science line t100 and Shimadzu UV-1600, respectively. The size and size distribution of the self-assembled structures were determined by dynamic light scattering (DLS) measurements on a Zetasizer Nano ZS (Malvern Panalytical). The morphology of the self-assembled structures was observed with transmission electron microscopy (TEM, JEM-2000FX), field emission scanning electron microscopy (FE-SEM, JSM-7600F), and optical microscopy (Hirox KH-8700 and A KEYENCE VHX-2000 equipped with a Mettler FP82 HT hot stage). X-ray diffraction (XRD) patterns were acquired on a Rigaku Ultima-IV system using Cu Kα radiation at 40 kV and 40 mA. Small angle X-ray scattering (SAXS) measurements were carried out at SPring-8 BL03XU equipped with a PILATUS 1M detector (Dectris®). The Fit2D program was used for the analysis.

### 2.3 Calcein release behavior

To investigate the photosensitivity of the vesicles that were spontaneously formed through the self-assembly of N6 and M6, encapsulated calcein was used as a probe. The calcein-encapsulating vesicles were prepared by mixing N6, M6, and calcein according to the method reported previously.^46^ Then, 30 μL of the calcein-encapsulating vesicles was mixed with 2.0 mL of Tris–HCl buffer (pH = 7.4) aqueous solution in a cuvette, which was in the holder of a spectrofluorometer (Hitachi F-7000) at 25 °C. The fluorescence intensity of calcein was monitored at 515 nm (excited at 495 nm) by fluorometry. To measure the fluorescence intensity corresponding to complete calcein release, 15 μL of 10% Triton X-100 aqueous solution was added to disrupt the vesicles. The percentage of calcein released was calculated with the reported previously equation:^46^100% × (*F* − *F*_0_)/(*F*_*t*_ − *F*_0_)where *F* is the fluorescence intensity obtained after light irradiation, *F*_0_ is the intensity observed for the fresh sample, and *F*_*t*_ is the intensity observed after treatment with 10% Triton X-100.

## 3. Results and discussion

### 3.1 Self-assembly behaviour

**Fig. 1:**
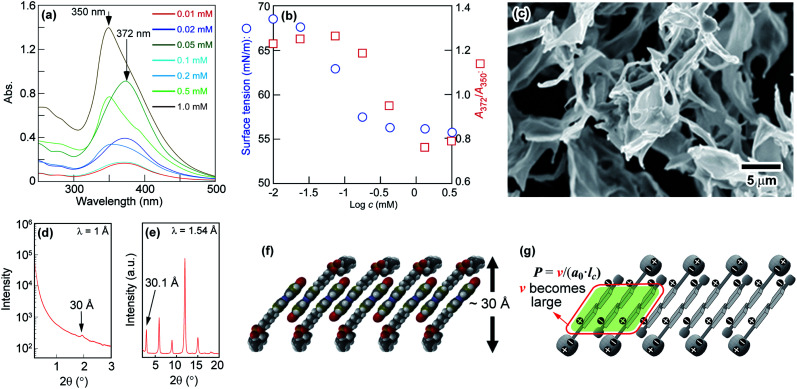
(a) Changes in the absorption spectra; (b) surface tension and *A*_372_/*A*_350_ as a function of N6 concentration in water; (c) FE-SEM image of freeze-dried N6; (d) SAXS profile of freeze-dried N6; (e) XRD profile of N6 powder; and (f and g) molecular packing models of the precipitated N6 assemblies in water.

To explore the self-assembly behaviour of N6 in water, the absorption spectra and surface tensions of N6 in water were measured. As shown in Fig. 1a, an absorption band at approximately 372 nm corresponding to the π–π* transition was observed at a lower concentration (*c*) in water (*e.g.*, *c* = 0.05 mM). The absorption band shifted to a shorter wavelength (350 nm) as the concentration was increased (*e.g.*, *c* = 1.0 mM). The blueshift of this band with increasing concentration was due to the formation of H-aggregates, and the driving force for this aggregation is further enhanced by the stacking of the Az moieties.^43,47,48^Fig. 1b shows the changes in the ratio between the absorption intensities at 372 nm and 350 nm (*A*_372_/*A*_350_) and the surface tension as a function of the N6 concentration in water. An abrupt change in *A*_372_/*A*_350_ as well as the surface tension was observed at approximately 0.1 mM, and these values plateaued when the concentration was increased beyond 0.5 mM. These results indicate that the self-assembly of these molecules into H-aggregates occurs at concentrations higher than 0.1 mM N6 in water, and the formation of H-aggregates is closely related to self-assembly.

The SEM images of freeze-dried N6 that had self-assembled in water at 2 mM showed that the particles had a sheet-like structure (Fig. 1c). As shown in Fig. 1d, a broad and weak scattering peak was observed in the SAXS data of the freeze-dried N6. This result suggested that freeze-dried N6 has a layer structure with an interlayer distance of *ca.* 30 Å, and the layer structure might be maintained in water. The XRD profile of the N6 crystalline powder shown in Fig. 1e revealed that N6 has a layer structure with an interlayer distance of 30.1 Å in the crystal state. The length of the N6 molecules estimated using Spartan was 28 Å, which is roughly equal to the thickness of the layer structure. However, the formation of a stable single layer in which N6 molecules are arranged in a parallel “head-to-head” manner is difficult since the surface consisting of the Az groups with no hydrophilic group is unstable at the water interface. Consequently, the layer structure is expected to be bilayered in order to form a stable interface with water. When the sheet-like structure is a simple bilayer with a cross section of two N6 molecules, the thickness of the bilayer should be approximately 56 Å. The extremely small thickness of the structure suggests that N6 molecules overlap in the bilayer to form a “head-to-tail” self-assembled structure with a thickness of 30 Å. To clarify the self-assembled structure of N6 molecules in water, we tried to prepare a single crystal of N6 for evaluation by X-ray single crystal analysis. Despite extensive efforts, a single crystal of N6 was not obtained due to its low crystallinity; however, we did obtain an X-ray single crystal structure of an N6 analogue having a shorter methylene spacer, N3, as shown in Fig. S1 and S2.† The X-ray single crystal analysis showed that the N3 molecules are antiparallel and give a tilted and interdigitated bilayer structure in the crystal. Therefore, a similar packing model, shown in Fig. 1f, could be proposed for the formation of a tilted and interdigitated bilayer structure through the antiparallel stacking of N6 in water at concentrations higher than 0.1 mM. The dipole moment of the model molecule in which the *p*- and *p*′-positions of the Az unit were substituted with nitro and methoxy groups, respectively, was estimated to be 7.6 D by Spartan, as shown in Fig. S3.† Thus, the dipole moments of the Az groups in N6 and N3 were both expected to be approximately 7.6 D. The dipole moment of the Az group favours an antiparallel molecular orientation.^42^ From the above result, the most plausible self-assembled overlapping bilayer structure was formed by the antiparallel tilting of N6 molecules, as shown in Fig. 1f. The illustration of the packing of N6 as an interdigitated bilayer is supported by both of the theories suggested by Israelachvili^49^ and Nagle^50,51^ (Fig. S4†). Israelachvili *et al.* reported that the molecular shape of amphiphiles is critically important for the morphology of the self-assembling structures formed in water. The packing parameter, which considers the volume and the critical length of the hydrophobic tails, is defined as follows:*P* = *v*/(*a*_0_ × *l*_c_)where *P* is the packing parameter, *v* is the volume of the hydrophobic tail, *a*_0_ is the area of the hydrophilic head group, and *l*_c_ is the critical length of the hydrophobic tail. In the cases of *P* < 1/3 and 1/3 < *P* < 1/2, the structures of the assemblies are expected to be spherical and rod-like micelles, respectively, and vesicle and sheet-like bilayers will be observed for 1/2 < *P* < 1 and *P* = 1, respectively.^49^ Theoretically, amphiphiles with two hydrophobic tails, such as dipalmitoyl phosphatidylcholine (DPPC) and dipalmitoyl phosphatidylethanolamine (DPPE), tend to form bilayers because of the large volume of the hydrophobic tails. Notably, here, the orientations of the amphiphilic molecules in the bilayers are different: tilted molecular packing for DPPC and non-tilted molecular packing for DPPE. Nagle reported that the packing of the amphiphilic molecules depends on the relative size of the areas between the polar head group and the hydrophobic tails of the molecules.^50,51^ In the case of DPPC, in which the hydrophilic head group is larger than the hydrophobic tail, tilted molecular packing is favoured because the attractive van der Waals forces are stronger, as shown in Fig. S4.† On the other hand, the hydrophobic tails of DPPE molecules can interact with each other without tilting because the head group and hydrophobic tails of DPPE are similar in size, leading to a non-tilted molecular orientation.^50,51^

Fig. 1g depicts the tilted and interdigitated bilayer structure N6, which takes on an antiparallel molecular orientation in water. N6 molecules behave as amphiphiles with two or three hydrophobic tails, and they interact with neighbouring Az skeletons in the self-assembled aggregates. They are expected to have shapes similar to DPPC and DPPE in the bilayer structure, and DPPC and DPPE tend to form bilayers because of the large volumes of their hydrophobic tails. Thus, the volume of the hydrophobic tails of N6 were increased, resulting in a larger packing parameter and the formation of a bilayer. However, the interacting hydrophobic tails of N6 molecules are smaller than the polar hydrophilic heads. To maximize the van der Waals forces between the hydrophobic tails, tilted molecular packing in the form of a bilayer is proposed for the aggregates of N6 formed in water (Fig. S4†). In addition, the interactions of the phosphocholine groups of N6 molecules influence its self-assembly, because neither H-aggregation nor self-assembly was observed for an N6 ammonium analogue with an ammonium group but no phosphocholine moiety as the hydrophilic part (shown in Fig. S5†). Furthermore, as shown in Fig. S2(d),† the phosphocholine moiety of N3 bent away from the long axis of the Az skeleton to increase the affinity for the water layer through hydrogen bonding. The bending behaviour of the phosphocholine moiety might enable phospholipids without a long alkyl tail to form a bilayer-type self-assembled structure through H-aggregation. Actually, it is impossible for the hydrophilic parts amphiphiles with only ammonium moieties but no phosphocholine moiety to bend in this way. According to the above results, the π–π stacking, the large dipole moment of the Az group, the attractive van der Waals forces between the hydrophobic moieties and the interactions and bending behaviour of the phosphocholine group drive the N6 molecules to aggregate and overlap in an antiparallel manner as a tilted and interdigitated bilayer.

**Fig. 2:**
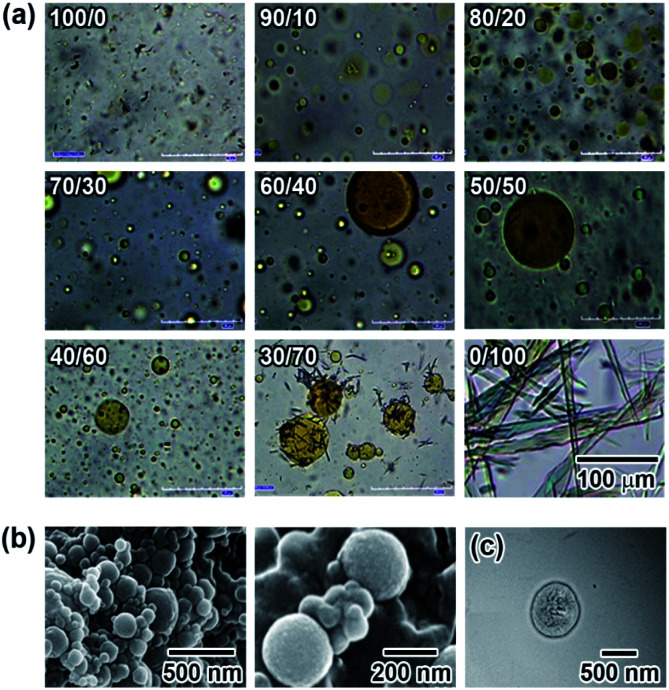
(a) Changes in the morphology of the self-assemblies formed by mixing N6 and M6 at different molar ratios. The total molar concentration of N6/M6 was adjusted to 2 mM. The scale bar in the lower right photo is applicable to all the photos in (a). (b) FE-SEM images of a freeze-dried mixture of N6 and M6 (*R* = 50/50) in water. (c) TEM image of a representative vesicle obtained by mixing with N6 and M6 (*R* = 50/50) in water. The image was taken after phosphotungstic acid staining.

Phospholipids having two hydrophobic fatty acyl tails form symmetric sheet-like bilayers.^14,49^ Furthermore, owing to the energetic instability of hydrophobic domains at the edge of the bilayer in water, the sheet-like bilayers tend to form a sealed structures where the aqueous inner and outer regions are separated by the resulting hydrophobic bilayer to give a spherical vesicle. However, for N6 in water, the relatively high rigidity and stability of the bilayer prevent it from further assembling into sealed vesicles. Therefore, we next studied the effects of M6 on N6 self-assembling behaviour. M6, which has a methoxy group and an ether linker at the *p*- and *p*′-positions of the Az moiety, is expected to have a lower dipole moment than N6. The rigidity of the self-assembled aggregate formed by N6 would decrease when it was mixed with M6 because weak intermolecular interactions between N6 and M6 molecules were assumed to be introduced into the rigid sheet-like structure. M6, which was carefully designed and synthesized, showed low solubility in water at room temperature. To generate a uniform mixture of M6 and N6 in water without a non-aggregated and fully dissolved state, 2 mM aqueous solutions of M6 and N6 were prepared by heating at 90 °C. Then, the resulting solutions were mixed together at different volume ratios, and one day later, the mixtures were observed by optical microscopy at room temperature. As shown in Fig. 2a, needle-like crystalline particles were obtained for M6 in water (N6/M6 = 0/100) owing to its poor solubility. In contrast, there were aggregated dispersed solids of N6 in the water (N6/M6 = 100/0) due to the formation of tilted and interdigitated bilayer structures, as mentioned above. Interestingly, a different molar ratio *R* (*R*: N6/M6) between N6 and M6 preferentially afforded a molecular assembly with a different number of dimensions. When *R* equalled 10/90 and 20/80, the mixtures favoured the formation of fibrous fine crystals. In the range of *R* = 40/60 to 90/10, the mixtures afforded spherical substances as observed by microscopy. DLS, SEM, and TEM measurements were used to characterize the spherical structures obtained from an equimolar mixture of N6 and M6 (*R* = 50/50). For the SEM observation, dry vesicles were prepared by the rapid cooling of the vesicle dispersion followed by freeze drying. The images shown in Fig. 2b revealed that the dry vesicles formed spherical assemblies with diameters of *ca.* 200 nm. This diameter was dramatically different from that of the vesicles observed in Fig. 2a. The vesicles shown in Fig. 2a might disassemble during the freeze-drying process. The results shown in Fig. 2a suggest that the vesicles have a wide size distribution in water. A similar result was also obtained by DLS because both large and small vesicles with mean particle sizes of 12 ± 1 μm and 396 ± 93 nm were observed. Fig. 2c shows a TEM image of a representative vesicle. No onion-like multilamellar structures were seen on the surface of the vesicle. Based on the SEM and TEM images shown in Fig. 2b and c, the assembled structures obtained by mixing N6 and M6 are spherical unilamellar vesicles.

Next, the surface tension and absorption spectra of the N6/M6 mixtures in water were measured to explore the assembly behaviours of the mixtures. As shown in Fig. S6,† a blueshift of the absorption wavelength owing to the H-aggregation of the Az groups was observed, and abrupt changes in both the absorption wavelength and surface tension were found at approximately 0.1 mM. The results indicate that the formation of vesicles is closely related to the formation of H-aggregates when the concentration of the N6/M6 mixture is over 0.1 mM, which is similar to the case of N6 as a single component in water. Furthermore, N6 and M6 were paired and arranged in a parallel manner owing to the weak intermolecular interactions (demonstrated in Fig. S7†) as well as the π–π stacking of the Az groups, and the paired N6–M6 would be linked to the N6 aggregate fragments to give an and interdigitated bilayer with some defects (shown in Fig. S8†). As the lengths of the N6 and M6 molecules were estimated to be 28 and 27 Å by Spartan, the thickness of the suggested bilayer model consisting of overlapping N6 and M6 molecules is expected to be approximately 38 Å, which is roughly coincident with the data found in the SAXS profile (38 Å, shown in Fig. S9†). Another reason for M6 to induce defects in the bilayer of the vesicles is that the estimated dipole moment of the Az group in the M6 molecule according to Spartan was 0 D (Fig. S3†), and M6 molecules do not necessarily pack with N6 or other M6 molecules into an antiparallel molecular orientation. Some N6 and M6 molecules would pack in a parallel manner, resulting in the formation of defects. Although it suggests that the number of defects is small (see Fig. S8†), it does contribute to the aggregation of N6 and M6 into a non-tilted bilayer. In addition, the interactions between N6 and M6 are not stronger than those among N6 molecules, even if N6 and M6 are packed in an antiparallel orientation. As a result, the addition of M6 would destabilize and increase the flexibility of the bilayer, causing the transformation from a bilayer into a spherical vesicle. The increase in the thickness of the bilayer from 28 to 38 Å is attributed to the destabilization and the increase in flexibility of the bilayers upon mixing M6 with N6.

### 3.2 Effect of photoisomerization on the morphology of vesicles

**Fig. 3:**
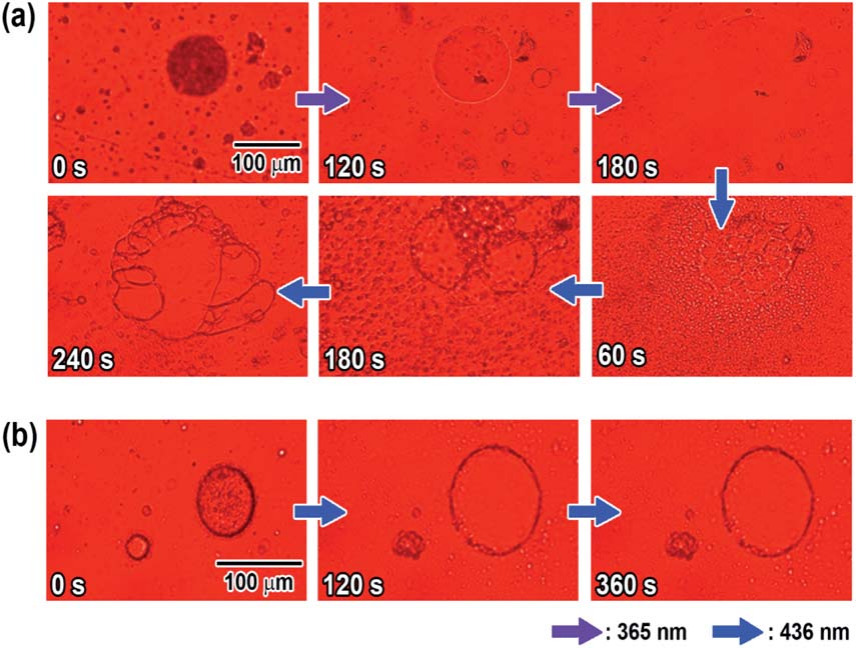
(a) Change in the morphology of the N6/M6 vesicles (*R* = 50/50, *c* = 2 mM) upon irradiation with UV light (365 nm, 16 mW cm^−2^) and then visible light (436 nm, 16 mW cm^−2^); (b) change in the morphology of the N6/M6 vesicles (*R* = 50/50, *c* = 2 mM) upon irradiation with visible light (436 nm, 16 mW cm^−2^).

Microscopic observation of the vesicles was carried out using UV and visible light irradiation to clarify the effects of the photoisomerization on the morphology of the N6/M6 vesicles in water. As shown in Fig. 3a, the vesicles with large diameters disappeared and decreased in size to a few tens of nanometers (as shown in Fig. S10†) upon irradiation with UV light at room temperature, while assemblies reappeared following visible light irradiation. The morphology was different from that before light irradiation. Fig. S12 and S13† show the changes in the absorption spectra and diameter of the vesicles as a function of UV irradiation time. Before UV irradiation, there was an absorption band at 340 nm corresponding to H-aggregation. UV irradiation caused a decrease and an increase in the absorbances at 340 nm and 450 nm, respectively, due to the photoisomerization of the Az groups in N6 and M6 from *trans* to *cis*, and the reverse situation was observed upon visible light irradiation. UV irradiation caused not only a change in the absorption spectra but also a change in the diameter of the spherical vesicles from 500 nm to a few tens of nm, as shown in Fig. S10.† Notably, irradiation with a longer wavelength of visible light can also result in a peculiar change in the morphology of the N6/M6 assembled structures, although the photochemical *cis*–*trans* reverse isomerization reaches a photostationary state under visible light irradiation for no longer than 30 s (shown in Fig. S12†). As shown in Fig. 3a, after irradiation by UV light, reassembled vesicles were obtained, and they kept changing their shape without reaching a stationary state during exposure to visible light for 180 s to 240 s. To determine the effect of visible light irradiation on the morphology of the N6/M6 vesicles, fresh vesicles were exposed to visible light for an extended time. Interestingly, a similar situation, the swelling of the vesicles formed by N6 and M6, was also found in Fig. 3b; the size of the vesicle kept increasing under continuous visible light irradiation. This result was confirmed by the DLS experiments (Fig. S13†).

UV irradiation changes the physical properties of the Az groups, such as their molecular shape and polarity. There are many reports on the morphological changes in self-assembled structures containing Az units due to *trans*–*cis* photoisomerization upon UV light irradiation. The 436 nm visible light, on the other hand, is absorbed by both the *trans* and *cis* forms, causing a *trans*–*cis*–*trans* photoisomerization cycle. Consequently, little change in the absorbance is generally observed upon visible light irradiation. In this study, little change in the absorbance was observed for the N6/M6 vesicles upon visible light irradiation (Fig. S14†). In the case of N6/M6 vesicles, one of the important driving forces for the formation of the vesicle is H-aggregation, which is related to the planarity of the Az groups. Therefore, continuous instant changes in the planarity of the Az groups due to the *trans*–*cis*–*trans* photoisomerization cycle is considered to weaken the intermolecular interactions between the molecules, leading to the disassembly or expansion of the vesicles.

**Fig. 4:**
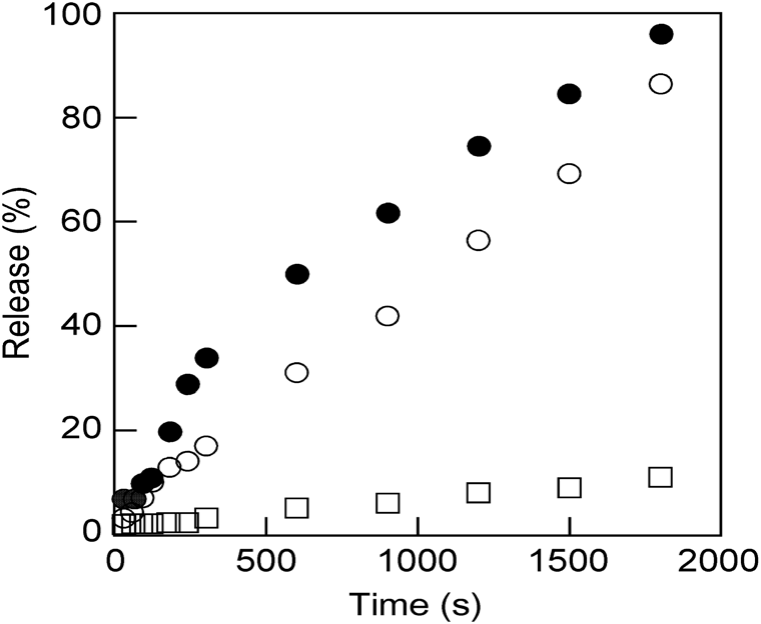
Calcein release behavior from the vesicles obtained by mixing M6 and N6 (*R* = 50/50, *c* = 0.5 mM) upon UV light (open circles: 16 mW cm^−2^) or visible light (filled circles: 16 mW cm^−2^) irradiation and without irradiation (open squares) at room temperature.

These nanosized vesicles, which can be obtained by mixing M6 to N6 in water (Fig. S11†), are very promising in the field of smart DDSs in response to external stimuli. Thus, N6/M6 vesicles (*R* = 50/50, *c* = 0.5 mM) encapsulating a fluorescence dye, calcein, were prepared by mixing N6, M6 and calcein according to the method reported previously,^46^ and the release behaviour of calcein was explored at 25 °C upon UV light or visible light irradiation. As shown in Fig. 4, only trace calcein was spontaneously released from the vesicles under dark conditions even over a long time, which indicated that the vesicles are stable at room temperature. However, calcein was dramatically released when the vesicles were exposed to either UV light or visible light. The size of the calcein-encapsulating vesicles was reduced by UV light but increased by visible light, as shown in Fig. S10 and S13.† Interestingly, the calcein was released faster under visible light than under UV light. As a consequence, visible light is a more effective stimulus for N6/M6 vesicles, even though the absorbance at 436 nm is weaker than that at 365 nm.

## 4. Conclusions

The investigation described above revealed that N6 aggregates in water pack into tilted and interdigitated bilayer where the Az units overlap in an antiparallel manner. These results also suggest that the parameter *R* has an important effect on the morphology of the aggregate and that an appropriate *R* leads to the M6/N6 mixture forming vesicles. A packing model for the obtained vesicles was suggested as a non-tilted and interdigitated bilayer, where the N6 molecules overlap in an antiparallel manner and M6 molecules are arranged on the sides of N6. The vesicles spontaneously formed through the self-assembly by N6 and M6 undergo an isomerization-driven morphological change upon UV and visible light irradiation. The calcein release behaviour from nanosized liposomes was observed upon irradiation with not only UV light but also visible light. The results of this investigation provide a unique strategy for designing visible light-sensitive carriers for DDSs because the use of UV light for the release of a drug in the human body is undesirable.

## Conflicts of interest

There are no conflicts to declare.

## Supplementary Material

RA-010-D0RA06803A-s001

RA-010-D0RA06803A-s002
