# Supplementary material for: Self-assembly of photoresponsive azo-containing phospholipids with a polar group as the tail
Source: RSC Adv. 2020 Sep 4;10(54):32984–91. doi: 10.1039/d0ra06803a (PMC9056651; doi:10.1039/d0ra06803a)
Supplement: RA-010-D0RA06803A-s001 [file RA-010-D0RA06803A-s001.pdf]

Electronic Supplementary Material (ESI) for RSC Advances.
This journal is © The Royal Society of Chemistry 2020

Electronic Supplementary Information

Self-assembly of Photoresponsive Azo-containing Phospholipids with a Polar Group as the Tail

Su Ma[a,b], Seiji Kurihara[a*], Yasuhiro Tomimori[a], Sunnam Kim[a], Eunsang Kwon[c], Atsushi Muramatsu[d], and Kiyoshi Kanie[d*]

[a]Department of Applied Chemistry and Biochemistry, Graduate School of Science and Technology, Kumamoto University, 2-39-1 Kurokami, Chuo-ku, Kumamoto, 860-8555, Japan.
E-mail: kurihara@gpo.kumamoto-u.ac.jp

[b]School of Chemistry, Biology and Material Engineering, Suzhou University of Science and Technology, No. 99 Xuefu Road, Huqiu district, Suzhou, 215009, China

[c]Graduate School of Science Research and Analytical Center for Giant Molecules, Tohoku University, 6-3 Aramakiazaaoba, Aoba-ku, Sendai, 990-8578, Japan

[d]Institute of Multidisciplinary Research for Advanced Materials, Tohoku University, Katahira 2-1-1, Aoba-ku, Sendai 980-8577, Japan
E-mail: kanie@tohoku.ac.jp

Table of contents

1. Characterization of **N3**, **N6** and **M6**
2. Crystal data and X-ray single crystal structure of **N3**
3. Calculation of dipole moment of azobenzene derivatives by Spartan
4. Consideration about sheet-like bilayer structure formation by van der Waals forces
5. Effect of hydrophilic moiety on the formation of *H*-aggregates
6. Changes in absorption spectra and surface tension by the change in the concentration of the mixture of **N6**/**M6** in water (***R*** = 50/50)
7. ^{1}H NMR characterization to form self-assembled vesicles in water
8. A plausible self-assembled bilayer structures by mixing **M6** to **N6**
9. SAXS characterization of freeze dried **N6**/**M6** mixtures in water with different mixing ratio
10. Changes in diameter of the **N6**/**M6** vesicles (***R*** = 50/50, *c* =2 mM) by irradiation with UV light
11. Changes in the absorption spectra of the vesicles as a function of irradiation time of UV light
12. Change in DLS profiles of **N6**/**M6** vesicles (***R*** = 50/50, *c* =2 mM) by visible light irradiation
13. Change in absorption spectra of the **M6**/**N6** vesicles (***R*** = 50/50, *c* = 0.5 mM) in water by irradiation of visible light

1. Characterization of N6, M6, and N3

NMR spectra were measured in CD_3OD solutions. ^1H NMR spectra were recorded on a JEOL JNM-LA400 at 400 MHz. The chemical shifts of the ^1H NMR signals were referenced to Me_4Si as the internal standard (δ = 0.00) and are expressed as chemical shifts in ppm (δ), multiplicity, coupling constant (Hz), and relative intensity. Elemental analyses were carried out by the Elemental Analysis Laboratory, Institute of Multidisciplinary Research for Advanced Materials, Tohoku University, using a Yanako MT-6 CHN Corder. The compounds N6, M6 and N3 were determined as follows:

N6: M.p. = 253.8-254.9 °C. 1H-NMR (400 MHz, CD3OD), δ (TMS, ppm): 8.39 (d, J = 9 Hz, 2H), 8.02 (d, J = 9 Hz, 2H), 7.96 (d, J = 9 Hz, 2H), 7.09 (d, J= 9 Hz, 2H), 4.28-4.23 (m, 2H), 4.11 (t, J = 6 Hz, 2H), 3.94 (tt, J = 6, 6 Hz, 2H), 3.62-3.60 (m, 2H), 3.21 (s, 9H), 1.87-1.81 (m, 2H), 1.73-1.66 (m, 2H), 1.54-1.48 (m, 4H). Elemental analysis: Calculated for C23H33N4O7P (+2H2O) (%): C, 50.73; H, 6.85; N, 10.29. Found (%): C, 50.74; H, 6.62; N, 10.27.

M6: M.p. = 239.7-241.9 °C. 1H-NMR(400 MHz, CD3OD), δ (TMS, ppm): 7.85 (d, J = 3 Hz, 2H), 7.83 (d, J = 3 Hz, 2H), 7.05 (d, J =3 Hz, 2H), 7.02 (d, J =3 Hz, 2H), 4.26-4.22 (m, 2H), 4.07 (t, J = 6 Hz, 2H), 3.93 (tt, J = 6, 6 Hz, 2H), 3.78 (s, 3H), 3.62-3.59 (m, 2H), 3.21 (s, 9H), 1.85-1.81 (m, 2H), 1.71-1.67 (m, 2H), 1.55-1.50 (m, 4H). Elemental analysis: Calculated for C24H36N3O6P (+2H2O) (%): C, 54.46; H, 7.62; N, 7.94. Found (+2H2O) (%): C, 54.44; H, 7.56; N, 7.93.

N3: M.p. = 243.1-244.8 °C. 1H-NMR (400 MHz, CD3OD), δ (TMS, ppm): 8.39 (d, J = 9 Hz, 2H), 8.02 (d, J = 9 Hz, 2H), 7.98 (d, J = 9 Hz, 2H), 7.13 (d, J= 9 Hz, 2H), 4.28-4.23 (m, 4H), 4.12-4.08 (m, 2H), 3.59 (t, J = 4 Hz, 2H), 3.19 (s, 9H), 2.17-2.14 (m, 2H). Elemental analysis: Calculated for C20H27N4O7P (+1.5H2O) (%): C, 48.68; H, 6.13; N, 11.35. Found (%): C, 48.63; H, 6.16; N, 11.25.

2. Crystal data and X-ray single crystal structure of N3

For the evaluation of the self-assembling structure of **N6** and **N6**/**M6** mixtures, x-ray single crystal structure analysis of **N3** was carried out. The single crystal was obtained by slow cooling of aqueous hot solution of **N3** containing acetonitrile. The crystal data were collected by a R-AXIS RAPID diffractometer using multi-layer mirror monochromated Cu-Kα radiation at 90 K, and the crystal structure was analyzed using the SHELXL within the OLEX-2 GUI for modelling the molecular structures. The refined data for **N3** are as follows: Triclinic, space group *P*-1 (No. 2), $(C_{20}H_{27}N_4O_7P)\cdot 3H_2O$; a = 8.3108(3), b = 11.6756(3), c = 25.7039(8) Å, α = 81.273(6) °, β = 88.301(6) °, γ = 83.153(6) °; V = 2447.51(14) Å^3; Z = 4; R = 0.1060, wR_2 = 0.2386.

Crystallographic data of **N3** has been deposited with Cambridge Crystallographic Data Center, deposition no. CCDC 2003927.

Figure S1 shows a molecular structure (ORTEP) and the single crystal structure of **N3** viewed along *a*-, *b*-, and *c*-axes.

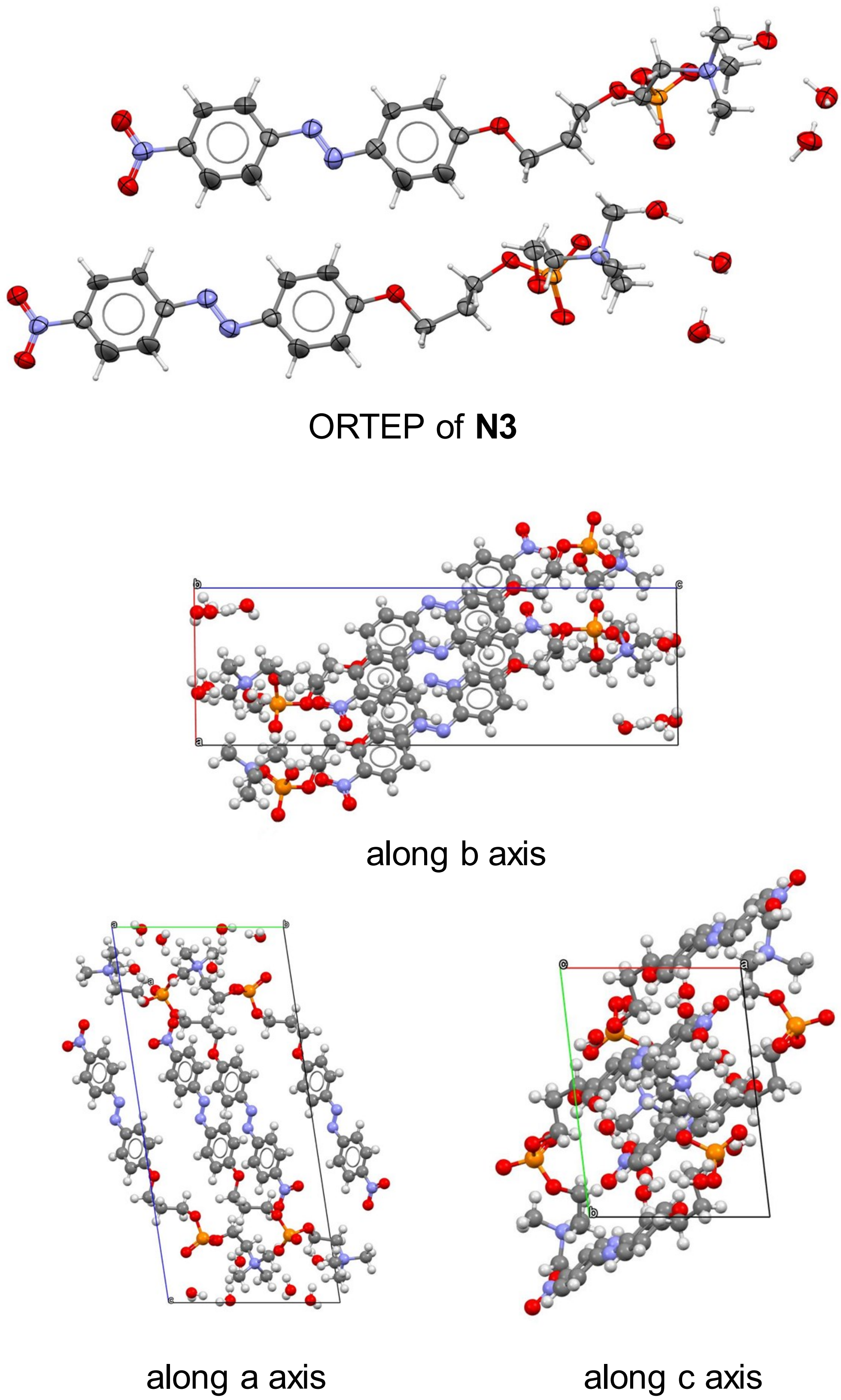

Figure S1. ORTEP diagram (thermal ellipsoids drawn at 50% probability) and An X-ray single crystal structure of **N3**.

Figure S2 (a), (b), and(c) shows the single crystal structure of **N3**, high lightened water positions in the crystal, viewed along *b*-, *a*-, and *c*-axes, respectively. Compound **N3** forms tilted bilayer structure through *H*-aggregation of the **Az** moieties. Yellow-colored spheres in the Figures represent water molecules in the crystal. The water molecules were occupied interlayer between the bilayers of the **N3** molecules. Figure 2(d) shows the part of the crystal structure of **N3** focusing on the phosphocholine-moiety to clarify the formation mechanism of the stacked bilayer of **N6**. The magenta-colored part represents the phosphocholine-moiety of N3. The part bended away from the long axis of the **Az** skeleton to increase the affinity for the water layer through hydrogen bonding. The bending behavior of the phosphocholine moiety might enable phospholipids without a long alkyl tail to form a bilayer-type self-assembled structure through *H*-aggregation. Actually, it is impossible for the hydrophilic parts amphiphiles with only ammonium moieties but no phosphocholine moiety to bend in this way.

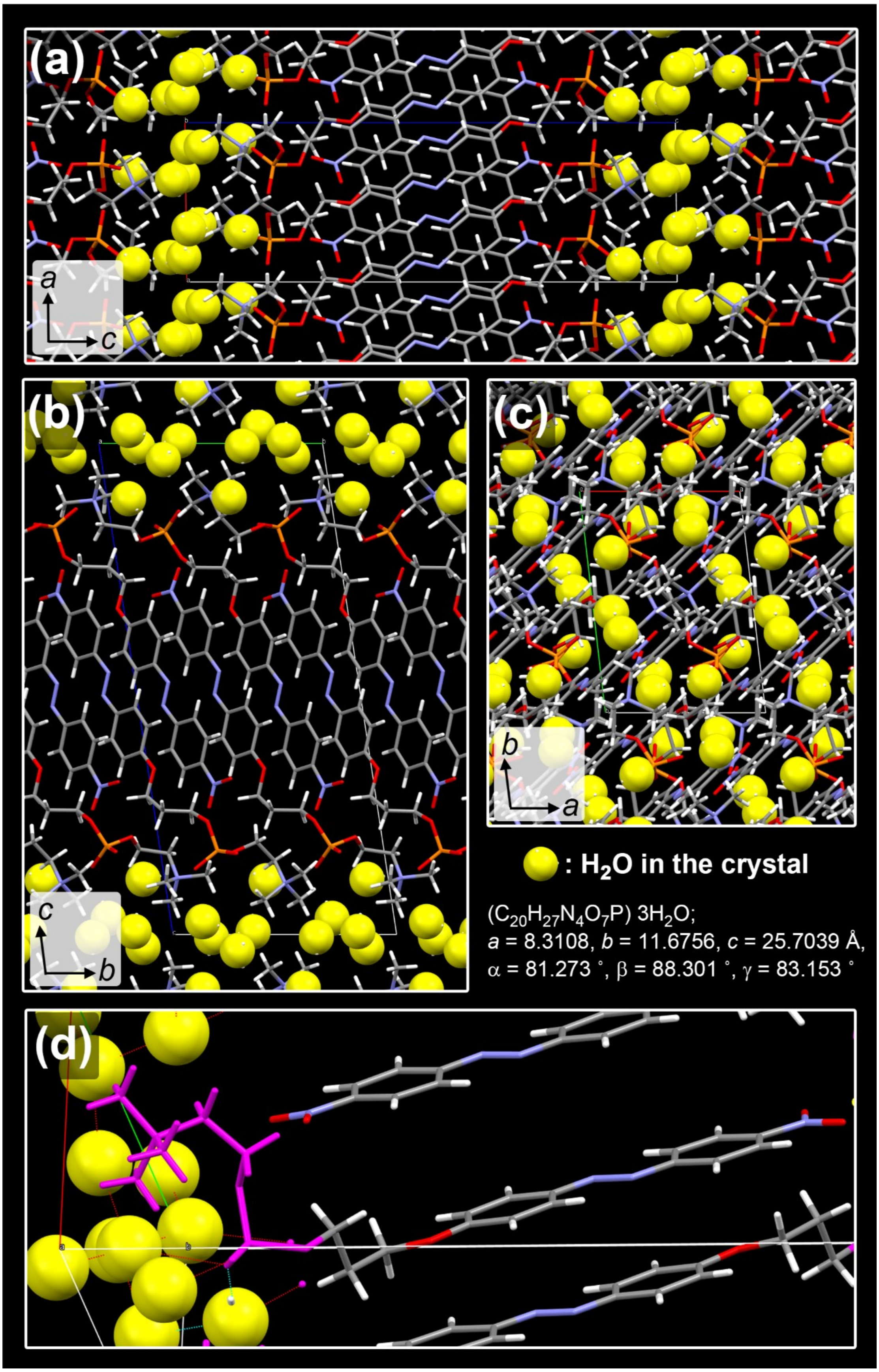

Figure S2. An X-ray single crystal structure of **N3**.

3. Calculation of dipole moment of azobenzene derivatives by Spartan

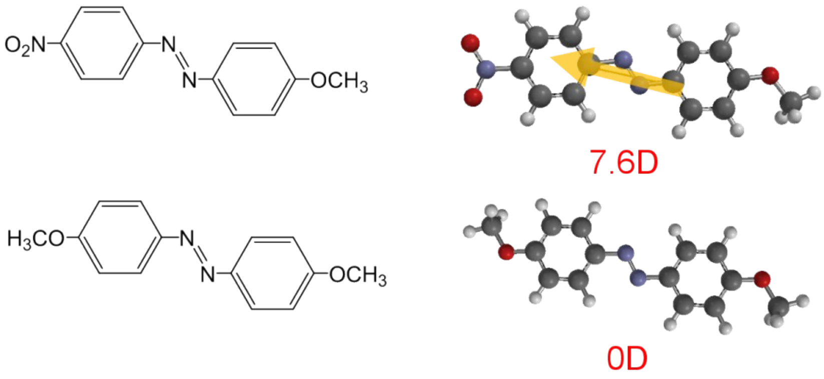

Figure S3. The dipole moment of **Az** compounds calculated by Spartan

4. Consideration about sheet-like bilayer structure formation by van der Waals forces.

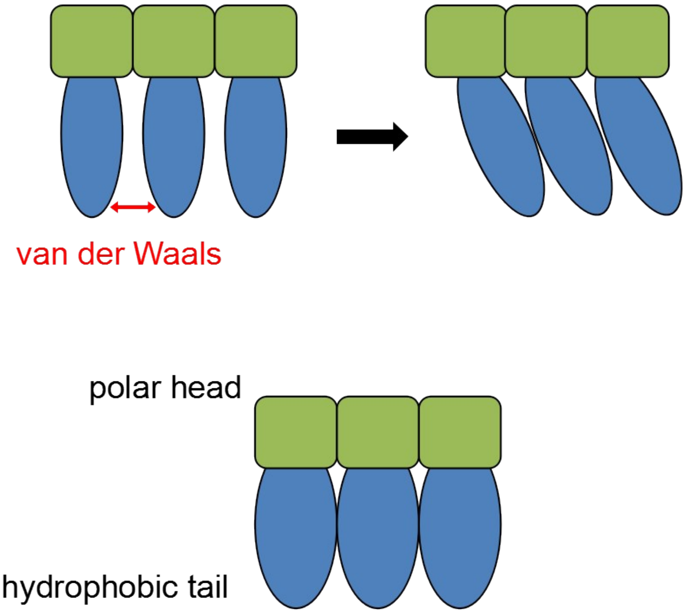

Figure S4. A schematic representation of the packing of lipid molecules with a polar head and a hydrophobic tail.

5. Effect of hydrophilic moiety on the formation of *H*-aggregates

As shown in Figure S5(a), the blue shift was not observed when the concentration of **NAz6TM** increased. It suggested that the **NAz6TM** molecules in water did not assembled into aggregation even at a higher concentration. As shown in Figure S5(b), the same situation was also found in the mixture of **NAz6TMA** and **MAz6TMA** in water.

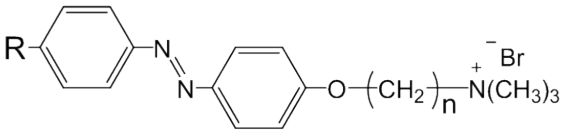

	R	n
MAz6TMA	CH3O	6
NAz6TMA	NO2	6

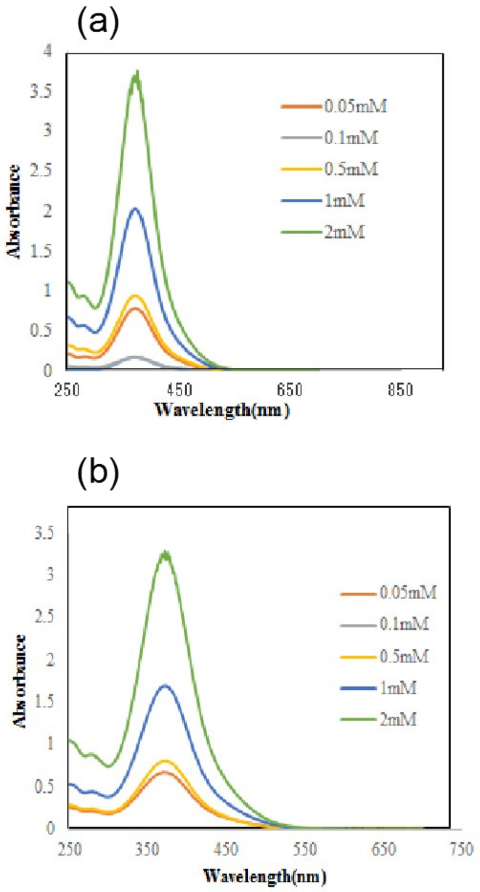

Figure S5. Absorption spectra of (a) **NAz6TMA**; (b) a mixture of **NAz6TMA** and **MAz6TMA** (***R*** = 50/50) in water

6. Changes in absorption spectra and surface tension by the change in the concentration of the mixture of N6/M6 in water (*R* = 50/50)

As shown in Figure S6(a), an absorption band at approximately 362 nm was observed at lower concentration in water (e.g. c = 0.05 mM). The wavelength of the absorption band was shifted to shorter wavelength (329 nm) by increasing the concentration (e.g. c = 1.0 mM). The blue shift of this band with increasing concentration was due to the formation of *H*-aggregates, and the driving force is furtherly enhanced by the stacking of the **Az** moieties. Figure S6(b) shows the changes in A_{362}/A_{329} and the surface tension as a function of concentration of **N6/M6** mixtures in water. An abrupt change in A_{362}/A_{329} as well as the surface tension was both observed at approximately 0.1 mM. These results indicate that self-assembly behavior of these molecules in *H*-aggregation occurs at concentrations higher than 0.1 mM of **N6/M6** mixture in water.

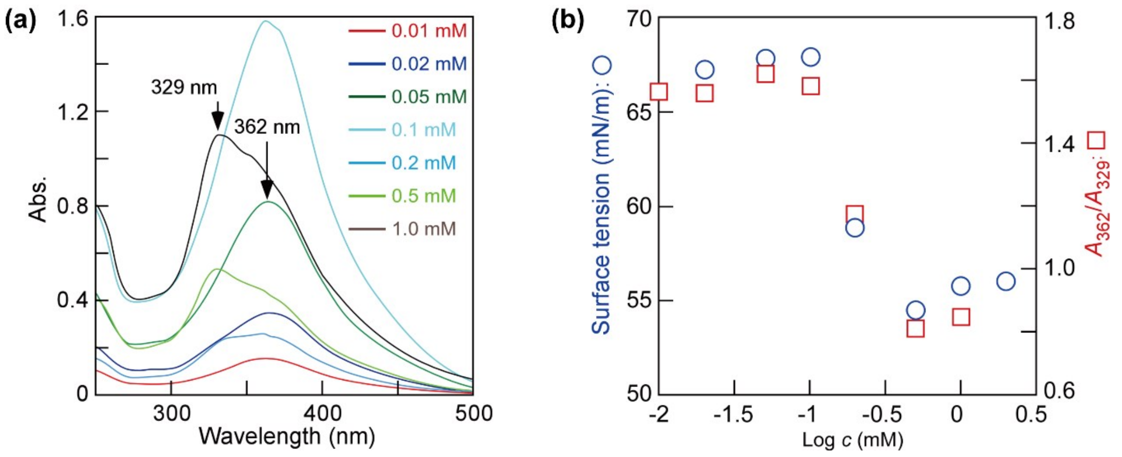

Figure S6. Changes in absorption spectra (a) and surface tension and A_{372}/A_{350} as a function of concentration of the mixture of **N6** and **M6** (***R*** = 50/50) (b) in water.

7. ^{1}HNMR characterization to form self-assembled vesicles in water

The solubility of **Az**-containing phospholipids is poor at room temperature, which resulted in the concentration of samples too low and signals too weak to be detected. Therefore, ^{1}H NMR measurements were performed at 60 °C in D_2O to investigate interactions between **N6** and **M6** (Figure S7). When measuring **M6** in D_2O at 60 °C using ^{1}H NMR, one of sharp peaks was obtained at 7.7 (δ/ppm), corresponding to the symmetrical aromatic hydrogens (-ArH-N=, 4H) (**M6**-a). Meanwhile, the peak at 7.8 (δ/ppm), was assigned to the aromatic hydrogens (O_2N-ArH-, 2H) (**N6**-a) of **N6**. However, a relatively broadened and shifted peak was observed at 7.51 (δ/ppm) for the **M6**-a and **N6**-a, when incubated **N6/M6** mixture in D_2O at 60 °C. The reason for the chemical shifts and broaden peaks was that electrostatic interactions was introduced between the groups MeO- and O_2N-. Consequently, the change of hydrogen environment drove the occurrence of chemical shifts and broadened peaks.

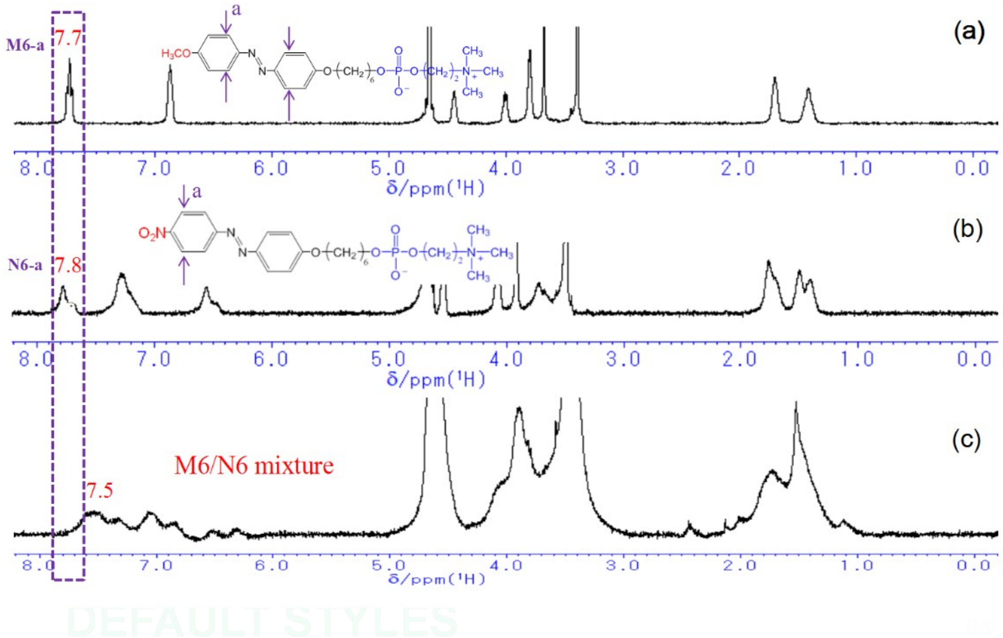

Figure S7. ^{1}H NMR of **M6** (a), **N6** (b) and **M6/N6** mixture (***R*** = 50/50, *c* = 2 mM) (c) in D_2O at 60 °C.

8. A plausible self-assembled bi-layer structures by mixing M6 to N6

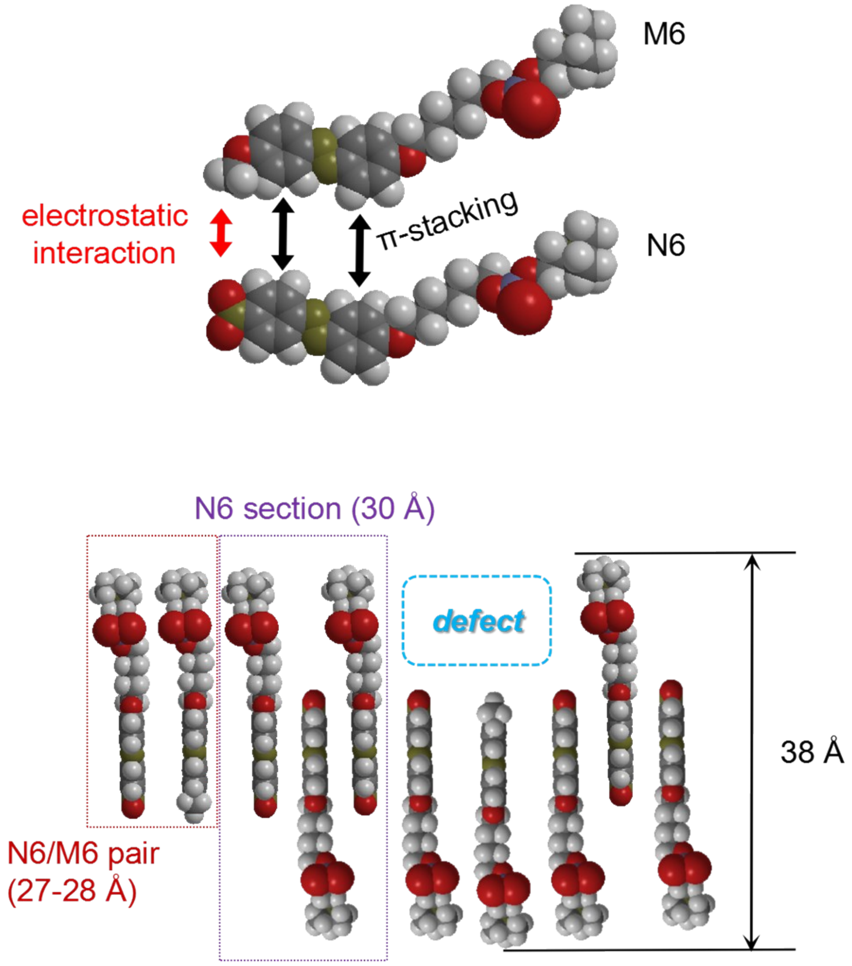

Figure S8. A plausible model for the molecular orientation of **N6** and **M6** (***R*** = 50/50) in the aggregation.

9. SAXS characterization of freeze dried N6/M6 mixtures in water with different mixing ratio

Figure S9 exhibits SAXS profiles of **N6**/**M6** mixtures prepared by freeze-drying from water dispersions. All the scattering profiles were taken at SPring-8 BL-03XU. Four peaks at 23, 27, 30, 38 Å were mainly observed, and the peak profiles were changed by the change in the **N6/M6** mixing ratio. By the comparison with the results shown in Figure 2, the scattering at 38 Å could be assigned as the bilayer thickness of the vesicles. On the other hand, peaks at 30 and 27 Å were due to layer thickness of **N6** and **M6** crystalline powders. As shown in Figure 2, there was neither phase separation nor recrystallization of **N6** and **M6**. In spite of careful treatment, freeze drying process might influenced the crystal formation. Further, an unassignable broad scattering at 23 Å were sometimes observed.

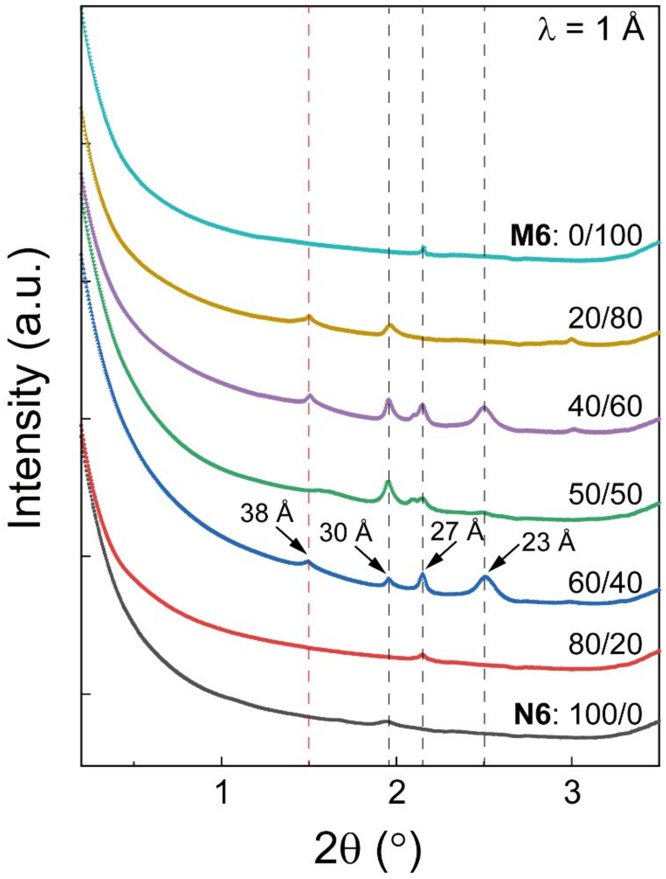

Figure S9. SAXS profiles of **N6**/**M6** mixtures prepared by freeze-drying from water dispersions. The total molar concentration of **N6** and **M6** in the dispersions was fixed to 2 mM.

10. Changes in DLS profiles of N6/M6 vesicles (*R* = 50/50, *c* =2 mM) by irradiation with UV light

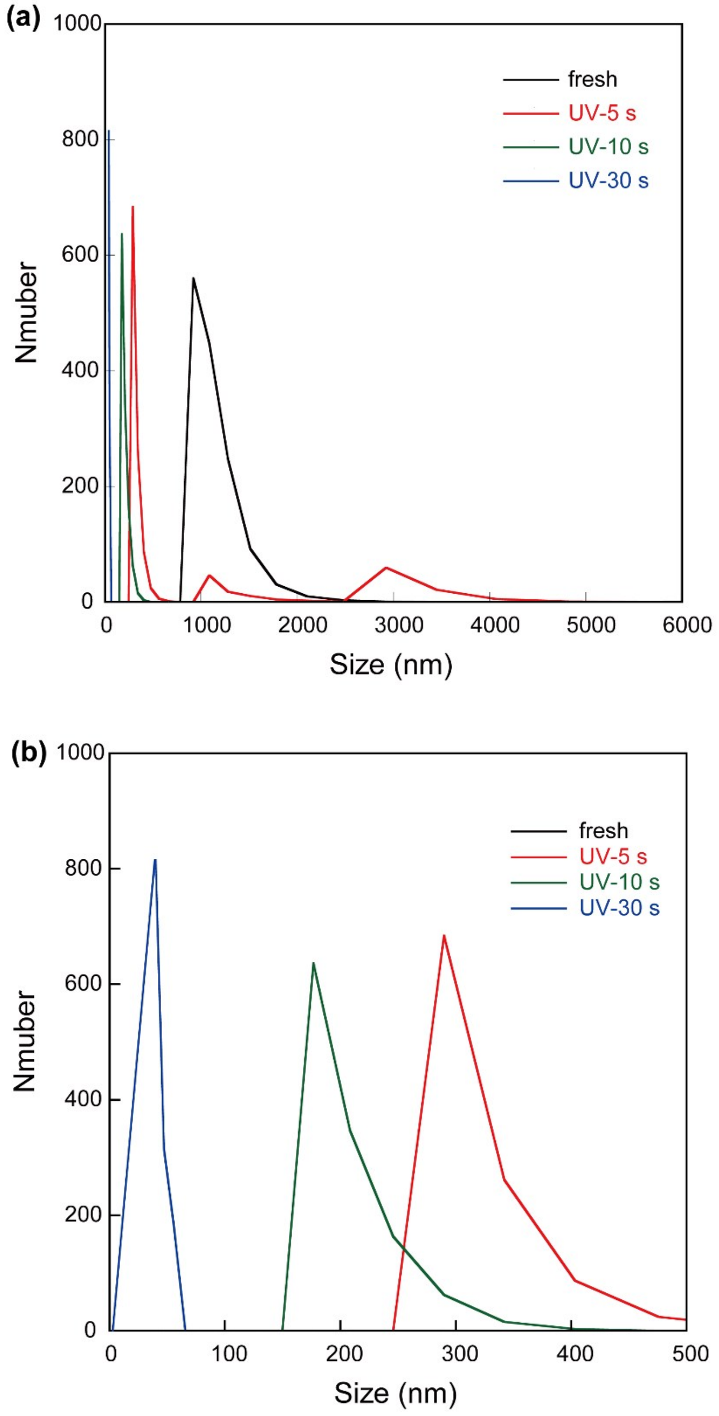

Figure S10. (a) Changes in DLS profiles of **N6**/**M6** vesicles (***R*** = 50/50, *c* =2 mM) by irradiation with UV light (365 nm,16 mW/cm^2); (b) the expanded image of (a).

11. Changes in the absorption spectra of the vesicles as a function of irradiation time of UV light

The **N6/M6** mixture (***R*** = 50/50, *c* = 0.5 mM) in water are vesicles with an average diameter of ca. 500 nm, which has been presented in Figure S11. Then, the changes in the absorption spectra of the vesicles as a function of irradiation time of UV light was detected. As shown in Figure S12, before UV irradiation, there was an absorption band at 340 nm, corresponding to the *H*-aggregation. The UV irradiation respectively caused the decrease and increase in absorbance at 340 nm and at 450 nm due to the photoisomerization of the **Az** groups in **N6** and **M6** from *trans*-form to *cis*-one, and the reverse situation was observed upon visible light irradiation less than 30 s.

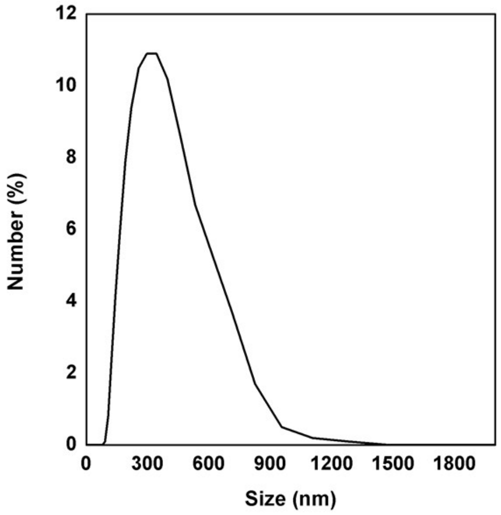

Figure S11. Average mean size of **N6/M6** vesicles (***R*** = 50/50, *c* = 0.5 mM) in water determined by DLS measurement.

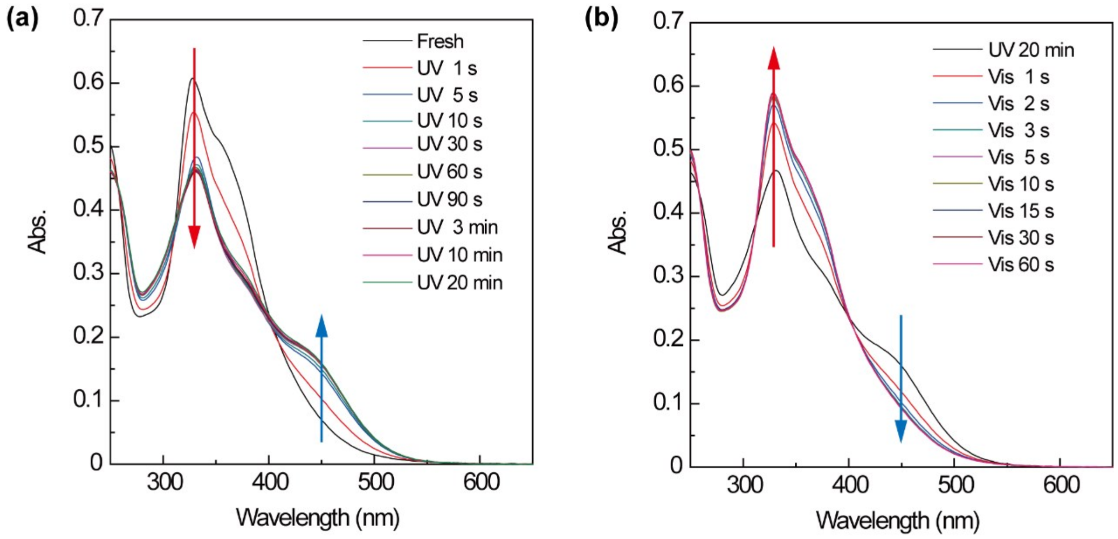

Figure S12. Changes in absorption spectra of the **N6/M6** mixture (R = 50/50, c =0.5 mM) in water by irradiation of UV light (365 nm, 1 mW/cm^2) and following visible light (436 nm,16 mW/cm^2).

12. Changes in DLS profiles of N6/M6 vesicles (*R* = 50/50, *c* =2 mM) by visible light irradiation

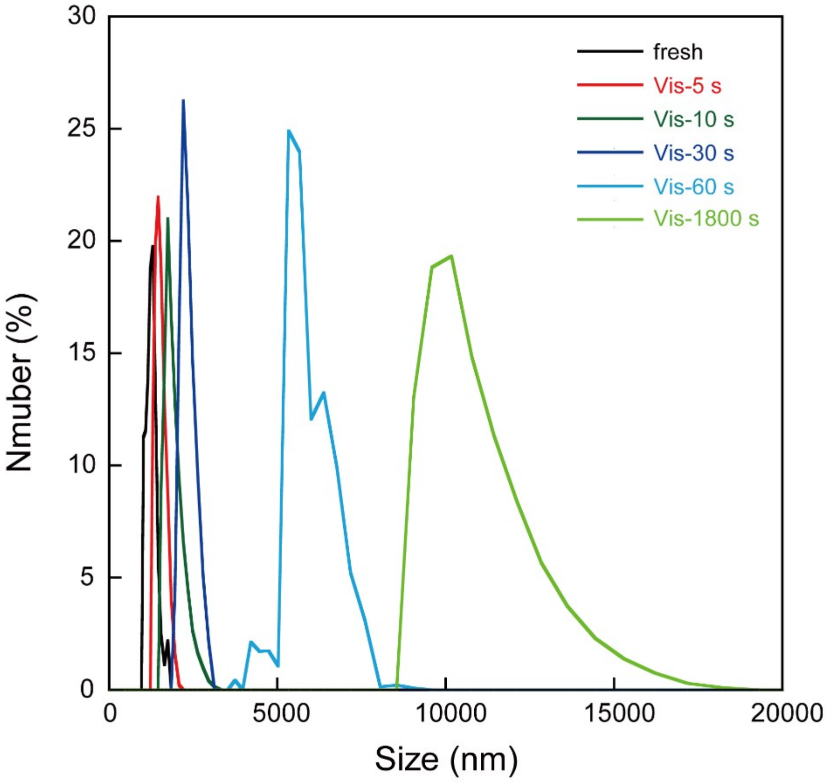

Figure S13. Changes in DLS profiles of **N6**/**M6** vesicles (***R*** = 50/50, *c* =2 mM) by visible light irradiation (436 nm, 16 mW/cm^2).

13. Changes in absorption spectra of the M6/N6 vesicles (*R* = 50/50, *c* = 0.5 mM) in water by irradiation of visible light

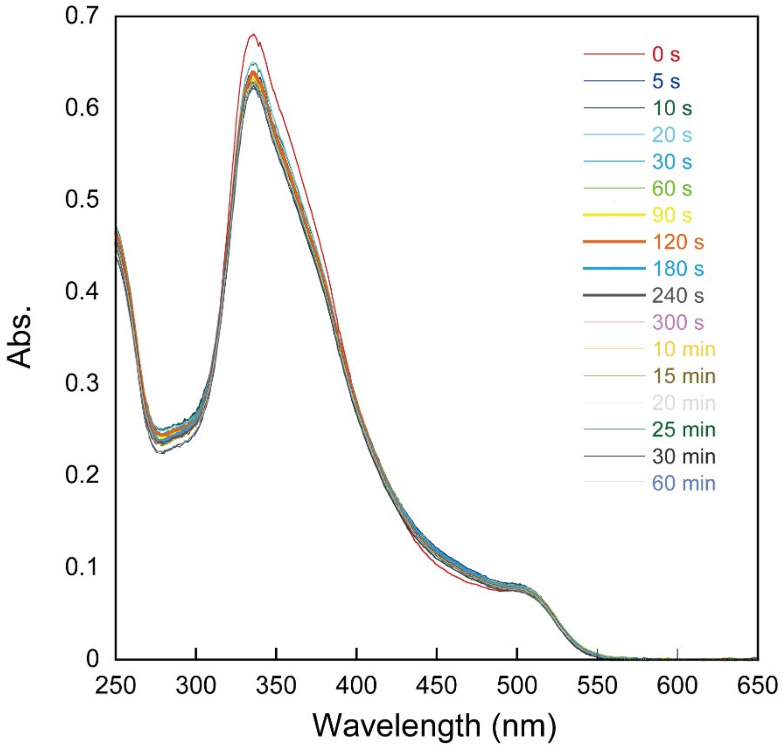

Figure S14. Changes in absorption spectra of the **M6**/**N6** vesicles (***R*** = 50/50, *c* = 0.5 mM) in water by irradiation of visible light (436 nm,16 mW/cm^2)
